# Research Progress in the Construction Strategy and Application of Superhydrophobic Wood

**DOI:** 10.3390/molecules30030719

**Published:** 2025-02-05

**Authors:** Siyu Chang, Lihong Yao, Lei Wang, Yueqi Wu

**Affiliations:** 1 College of Materials Science and Art Design, Inner Mongolia Agricultural University, Hohhot 010018, China; csy792111@emails.imau.edu.cn (S.C.); wxx@emails.imau.edu.cn (Y.W.); 2 Inner Mongolia Autonomous Region Russian and Mongolian Imported Wood Processing and Utilization Engineering Technology Research Center, Hohhot 010018, China

**Keywords:** superhydrophobic wood, durability, self-healing, coating material

## Abstract

Wood serves as a green biomass material with sustainable utilization and environmental friendliness. The modification of wood can be used to obtain superhydrophobic properties and further expand wood’s application range. This paper focuses on the development status of superhydrophobic surfaces with micro-/nanoscale rough structures. Based on the surface wettability theory, this paper introduces common methods of superhydrophobic modification of wood materials, compares the advantages and disadvantages of these methods, discusses the relationship between the surface microstructure and wettability, and summarizes the applications of superhydrophobic wood in oil–water separation, self-cleaning, and self-healing. Finally, the future development strategies of superhydrophobic coating materials are elucidated to provide basic theoretical support for the synthesis and diverse applications of superhydrophobic wood and a reference for subsequent research and development.

## 1. Introduction

Wood is a natural biomass material composed of cellulose, hemicellulose, and lignin, and it offers the advantages of wide availability, renewability, low cost, environmental friendliness, and biodegradability [1,2]. Owing to these characteristics, wood is widely used in fields such as furniture manufacturing, architecture, shipbuilding, and railway construction, which has aroused great interest in terms of the high-value utilization of wood resources [3,4]. However, wood exposed to humid environments absorbs moisture and water vapor, which causes wood deformation and decay as well as decreased strength, thereby reducing wood service life and ultimately limiting wood applications [5,6]. The strong affinity of wood for water stems from the abundant hydroxyl functional groups in hemicellulose and cellulose. Hydroxyl groups endow materials with good hydrophilicity, which have applications in fields such as drug delivery [7]. Meanwhile, materials with good hydrophobicity are often required in self-cleaning, oil–water separation, and other applications [8,9,10]. Therefore, constructing micro-/nanoscale rough structures on wood substrates and their superhydrophobic treatment can effectively resist liquid water infiltration [11,12], solving problems such as wood deformation, decay, and discoloration caused by moisture absorption [13,14]. The realization of superhydrophobic wood is important for the expansion of the application range of wood, extension of its service life, and achievement of its high-value utilization [15]. With the rapid development of micro-/nanotechnology, surface modification strategies are constantly emerging. Functional coatings synthesized by adding polymers can endow a substrate with excellent hydrophobicity and durability [16]. Superhydrophobic wood modified with titanium dioxide shows a certain level of flame retardancy, ultraviolet (UV) resistance, and antibacterial properties [17]. Through the addition of metal oxides, issues such as decay and insect infestation in superhydrophobic wood can be prevented [18]. Zinc oxide can be used to improve the UV resistance of wood [19]. The application of nanomaterials or other functional materials to modify superhydrophobic wood can enhance wood performance, meet stricter application requirements, and expand wood’s application range.

Inspired by natural superhydrophobic phenomena, the design of superhydrophobic structures has been extensively investigated in recent years. Notably, in the biological evolution process, different structures can lead to various characteristics and functions [20]. The “lotus leaf effect” is the most typical natural superhydrophobic phenomenon, and a poet once described the self-cleaning function exhibited by lotus leaves as “emerging from mud without staining [21,22]”. In addition, many other organisms, such as mussels, butterflies, water striders, roses, and peacocks, exhibit superhydrophobic phenomena [23,24,25]. The unique wetting characteristics exhibited by these organisms result from their continuous evolution to better adapt to their living environment. Research on superhydrophobic properties reveals that precisely controlling the micro–nanostructures of coatings is the key to synthesizing functional superhydrophobic coatings. In 1997, Bartholott and Neinhuis first observed the unique dual-scale layered micro-/nanostructure of lotus leaves under a microscope, which mainly comprises a microstructured mastoid and wax-like nanostructures on the mastoid. The layered structure with micro-/nanostructures constitutes a superhydrophobic surface with large contact angles and small rolling angles. Studies have shown that the surface morphology of lotus leaves exhibits a close relation with their superhydrophobic properties [26,27]. Inspired by the superhydrophobicity and self-cleaning ability of lotus leaves, Lv et al. prepared a Janus dual-function evaporator, which showed excellent photothermal conversion capability and achieved a long-term water source purification function, solving the problem of pollutant accumulation in water [28]. Ma et al. prepared a Janus film with excellent waterproofing using soybean polysaccharides, gelatin, and Brazilian palm wax. The Janus film displayed excellent antibacterial activity, antioxidant properties, and UV-blocking properties and was successfully applied to food packaging [29]. In addition to the “lotus leaf effect”, the “petal effect” is very famous [30]. The difference between the two is the different adhesion of leaves and petals to water. The “petal effect” involves higher water adhesion strength [31]. After flipping the petals, water droplets still adhere to the petal surface and do not easily roll off. The “lotus leaf effect” shows the exact opposite result. Rose petals show the most representative example of the “petal effect”. Inspired by rose petals, Zhang et al. used natural rubber latex and graphene–oxide liquid crystals as raw materials to obtain aerogels that were ultralight, tough, highly compressed, and sandwich-shaped via agitation and mixing [32]. Oopath et al. successfully replicated the rose-petal structure on the surface of a polyurethane acrylic film using nanoimprint lithography technology and UV-curing technology. The replicated surface exhibited same the water pinning effect as rose petals [33]. It can be observed that the synergistic effect of micro-/nanostructures and low-surface-energy substances is necessary for the construction of superhydrophobic surfaces [34]. Although superhydrophobic coatings have strong application advantages, various modifiers can induce different interfacial reactions during their use. Therefore, choosing the appropriate preparation process is the key to developing superhydrophobic coatings. In addition, coatings have low durability, further limiting the development of superhydrophobic coatings. Attention should be paid to ensuring adhesion between the substrate and coating, as poor adhesion can lead to the detachment of superhydrophobic coatings. The preparation of superhydrophobic coatings with high adhesion is reported in the literature [35,36].

Although superhydrophobic wood exhibits excellent superhydrophobic properties, there are challenges in its practical applications. Superhydrophobic coatings on wood surfaces often become fragile due to physical wear, chemical erosion, and temperature changes, leading to a weakening or even a loss of superhydrophobic properties. Therefore, the durability and chemical stability of coatings are crucial for the application of superhydrophobic wood. In addition, superhydrophobic coatings should not only meet the characteristics of nontoxicity and environmental protection but also demonstrate multifunctionality in some special scenarios, such as oil–water separation, self-cleaning, flame retardancy, self-healing, and anticorrosion [37,38].

In this review, inspired by biological surface wettability, the effect of surface structural parameters on properties was explored from the perspective of the structure and chemical composition of biological surfaces, and the findings provided templates and ideas for the preparation of biomimetic superhydrophobic surfaces and the control of interface mechanical behavior. Various preparation methods for superhydrophobic coatings were explored in detail, summarizing the latest applications of superhydrophobic-wood-based materials in different aspects and thus aiming to lay a foundation for a deep understanding of superhydrophobic wood. By endowing wood with superhydrophobic properties, the application range of wood can be broadened, its service life can be extended, and its high-value utilization can be achieved.

## 2. Superhydrophobic Surfaces

**Figure 1:**
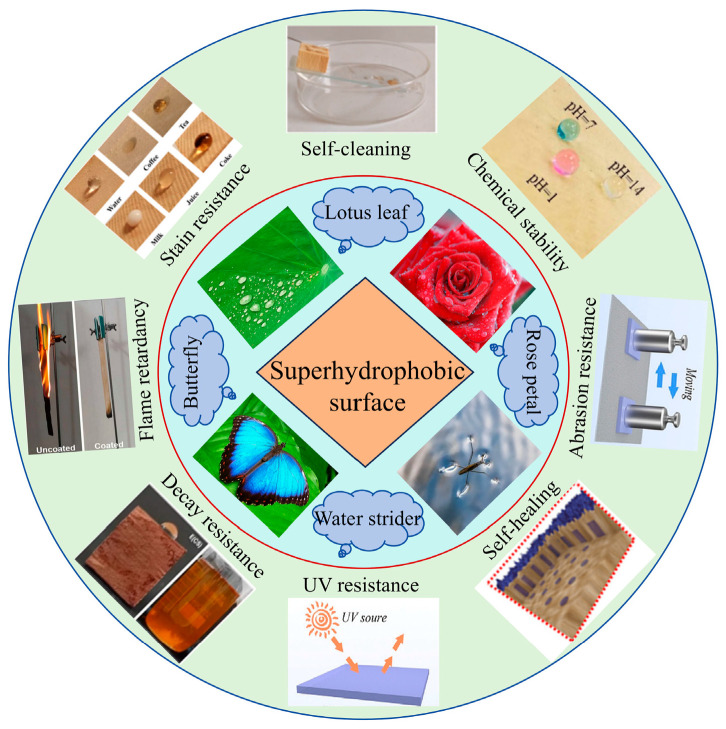
Superhydrophobic surfaces and their applications [43,44,45,46,47].

A superhydrophobic surface has special wettability, which is characterized by the formation of spherical water droplets on the superhydrophobic surface (Figure 1). Wettability refers to the contact between liquids and solid surfaces, which results from intermolecular interactions among gas, liquid, and solid phases [39]. Material surfaces exhibit wetting, which is usually measured using the contact angle, as a result of their low surface energy and microscopic roughness [40]. When the contact angle of a material is greater than 150° and it is wetted by water in a heterogeneous wetting regime (an advancing contact angle greater than ~150°, a roll-off angle of less than ~5°, or contact angle hysteresis of less than ~10°), the material surface is in a superhydrophobic state. When the material surface is completely covered with water and the contact angle reaches 0°, the surface exhibits a superhydrophilic state. The hydrophobic mechanism of materials is described in the literature [41,42].

### Basic Theory of Superhydrophobicity

**Figure 2:**
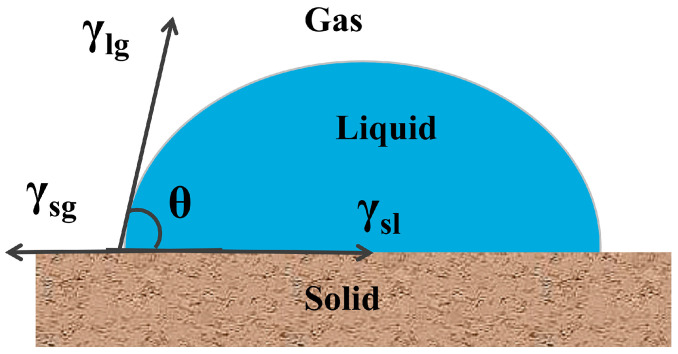
Young’s wetting equation and the force analysis of droplets on smooth surfaces.

The fundamental theory of surface wetting phenomena is elucidated here to investigate superhydrophobicity and superhydrophobic surface structures. After conducting extensive research on wetting phenomena, Young proposed the famous Young’s wetting equation in 1805, which describes the wetting behavior of droplets on a smooth, uniform, and ideal solid surface [48,49]. A contact angle is formed when a liquid comes into contact with a solid surface. The contact angle formed by water droplets on the surface is determined by interfacial tension at the solid, liquid, and gas three-phase contact surface and is independent of system geometry and gravity [50,51]. The contact angle (*θ*) is described as follows (Figure 2):

(1) $$cos\theta =\frac{\gamma_{sg}-\gamma_{sl}}{\gamma_{lg}}$$

where γ_sg_ represents the surface tension between solids and gases, γ_sl_ denotes the surface tension between solids and liquids, and γ_lg_ indicates the surface tension between liquid and gas.

**Figure 3:**
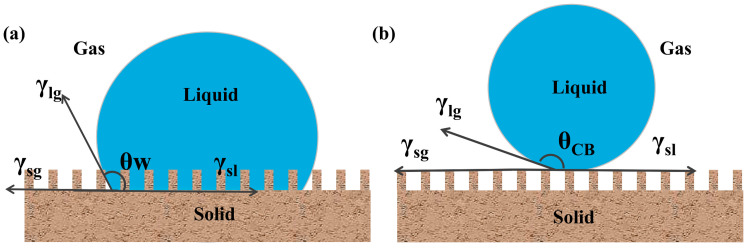
(**a**) Wenzel model, (**b**) Cassie–Baxter model.

The contact angle represents the wetting degree on a solid surface (Figure 2 and Equation (1)) [52]. Notably, *θ*_y_ = 0° indicates that a liquid is completely spread on a solid surface and attains a fully wetted state. Further, 0° < *θ*_y_ < 90° implies that the liquid can wet the solid surface. The smaller the *θ*_y_ value—which indicates hydrophilicity—the better the wettability. Meanwhile, 90° < *θ*_y_ < 150° indicates that the solid surface shows a water-repellent state. Further, for large *θ*_y_ values, the solid surface is less likely to be wetted. Moreover, 150° < *θ*_y_ < 180° indicates that the solid surface shows an evident water repellency. Large *θ*_y_ values indicate extreme difficulty in wetting the solid surface, which almost shows a superhydrophobic state. Finally, *θ*_y_ = 180° implies that the solid surface is not wetted, and the liquid appears completely spherical on the solid surface. Young’s wetting equation has its limitations and only applies to ideal surfaces that are smooth and isotropic with uniform chemical composition. However, such an ideal smooth and inert surface does not exist in reality [53,54,55]. All surfaces have a certain degree of roughness at the microscale level. In addition, as per Young’s wetting equation, the wettability of a solid surface can be changed only through the alteration of the surface free energy [56,57]. Research has shown that the contact angle of water droplets on a smooth surface with the lowest surface energy can reach only 120°, which is inconsistent with the actual situation in which the contact angle on superhydrophobic surfaces is observed to be greater than 150°. Therefore, Young’s wetting equation cannot be used to accurately explain the wetting behavior in practical conditions [58,59]. The micro-/nanomorphology of substrate surfaces has a crucial effect on their wetting behaviors during practical applications. Therefore, the rough structures and surface morphologies of solid surfaces need to be considered. Two main models are currently used for studying superhydrophobic phenomena on rough surfaces: the Wenzel and Cassie models (Figure 3) [60,61]. Wenzel believed that when a liquid comes into contact with a solid surface, it completely fills the grooves on the rough surface (Figure 3a). Roughness enhances the hydrophilicity of hydrophilic solid surfaces and the hydrophobicity of hydrophobic solid surfaces. The improved model is expressed using Equation (2):

(2) $$cos\theta_{w}=\gamma (\gamma_{s-g}-\gamma_{s-l})/\gamma_{l-g}=\gamma cos\theta$$

where *θ_w_* denotes the contact angle of a rough surface and γ indicates the surface roughness constant (the ratio of the actual area of solid–liquid contact and the projected area). A completely smooth solid surface has an actual area equal to the projected area, i.e., γ = 1. For rough surfaces, the actual area is greater than the projected area, i.e., γ > 1, due to their unevenness.

A solid surface exhibits hydrophilic properties when *θ* < 90°. As the surface roughness increases, the contact angle gradually decreases. In addition, surface roughness increases surface hydrophilicity. When *θ* > 90°, solid surfaces exhibit hydrophobic properties. As the surface roughness increases, the contact angle increases. Moreover, surface roughness increases the hydrophobicity of hydrophobic surfaces. Research has revealed that most superhydrophobic phenomena in nature, such as the “lotus leaf effect”, still cannot be explained using the Wenzel model [62]. The Wenzel model suffers from limitations, but it is suitable for modeling a uniform wetting state where a liquid completely occupies micro-/nanoscale rough structures on a solid surface [63,64]. Cassie and Baxter proposed a model for the nonuniform wetting state (Figure 3b). In this state, there is no direct contact between a liquid and a solid surface, and it is blocked by discontinuous air layers [65]. Equation (3) shows the Cassie–Baxter equation for determining the contact angle:

cos*θ*_CB_ = *r*_f_*f* cos*θ*_y_ + *f* − 1 (3)

where *θ*_CB_ represents the contact angle of a rough surface, *r*_f_ indicates the roughness ratio of the wetted area, and *f* represents area ratios of a solid surface wetted by a liquid.

In general, the wetting characteristics of a solid–liquid contact surface do not completely conform to a certain model; they show mixed conformity to the Wenzel and Cassie–Baxter models. In addition, changes in the surface roughness of solids can cause variations in the wetting characteristics, leading to the characteristics conforming interchangeably to the two wetting models [66]. A detailed analysis of the Wenzel equation and Cassie–Baxter equation can be found in a seminal work by Marmur [67]. In addition to the contact angle, two important characterization parameters are used for quantifying the wetting degree of solid surfaces: contact angle hysteresis and rolling angle [68,69]. Unlike the contact angle, contact angle hysteresis reflects the ease of droplet rolling when a liquid comes into contact with a solid surface. Wetting hysteresis is calculated as the difference between the advancing and receding contact angles measured when the speed of a three-phase (substrate/liquid/vapor) contact line tends to zero. A small difference indicates that a droplet is prone to rolling off, whereas a large difference indicates that the droplet exhibits strong adhesion and does not easily roll off. A low contact angle hysteresis is necessary for achieving the surface self-cleaning effect in materials. When a solid tilts to a certain angle, droplets on its surface slide or roll off [70,71]. The rolling angle refers to the minimum angle of inclination required for achieving droplet sliding or rolling. The measurement method for the rolling angle is simple, and superhydrophobic surfaces with a rolling angle of less than 10° are generally believed to have good self-cleaning capability [72,73]. The Wenzel and Cassie–Baxter models provide a strong theoretical basis for the preparation of superhydrophobic surfaces, and researchers have developed many techniques for the development of superhydrophobic films based on basic models.

## 3. Preparation of Superhydrophobic Wood

Wood is a biomass material with a natural rough structure on its surface, which is composed of protruding cell walls and grooved cell cavities. It can form multiple layers of rough structures together with nanoparticle (NP) clusters, which establish conditions necessary for the construction of superhydrophobic interfaces [74,75]. Two methods are used for preparing superhydrophobic wood surfaces: the first method involves the modification of wood using superhydrophobic materials and the construction of micro-/nanoscale rough structures on the wood surface. The second method involves the modification of rough structures using low-surface-energy substances, such as organosilanes, stearic acid, and perfluoro polymers. Given the anisotropy of wood, it is necessary to consider its unique structure and surface morphology during modification to avoid damaging the fiber morphology and texture of its surface, which may affect its aesthetics and usability [76,77]. Wood has a complex chemical composition, and achieving superhydrophobicity in wood necessitates appropriate methods and processes. Considering involved operation steps, preparation cost, application scope, and preparation conditions, current methods commonly used to prepare superhydrophobic wood include sol–gel [78], hydrothermal [79], template [80], spraying [81], and chemical vapor deposition methods [82].

### 3.1. Sol–Gel Method

**Figure 4:**
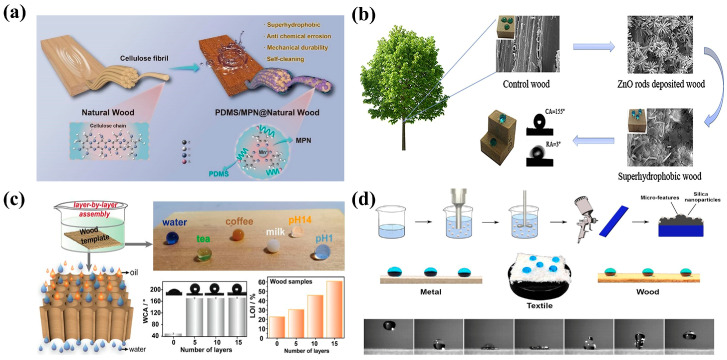
(**a**) Synthesis of superhydrophobic coatings on wood via the sol–gel method [84]; (**b**) hydrothermal synthesis of block-shaped superhydrophobic wood [90]; (**c**) preparation of superhydrophobic composite materials using wood as a biological template [99]; (**d**) synthesis of superhydrophobic coatings with excellent hydrophobicity and durability through the spray-coating method [110].

The sol–gel method is used to prepare superhydrophobic surfaces through the impregnation of NPs in a solution onto substrate surfaces. This process mainly uses compounds containing highly chemically active ingredients as precursors, which are subjected to hydrolysis, alcoholysis, or polymerization under normal temperature and pressure to obtain a uniform and stable transparent sol system. Then, the sol system is condensed into a gel with a three-dimensional spatial network structure. During treatment to achieve superhydrophobicity, wood is soaked in a superhydrophobic composite coating material, and a series of hydrolysis and condensation reactions are carried out to generate nanoscale rough structures on the wood surface. The sol–gel method shows good stability and has been developed rapidly in recent years. To improve the hydrophobicity of wood, Qu et al. used methyl triethoxysilane (MTES) and tetraethoxysilane (TEOS) as reaction precursors to prepare superhydrophobic coatings on a wood surface via the sol–gel process. The influence of the TEOS: MTES molar ratio on the coating performance was analyzed during synthesis, and the relationship between the coating structure and performance was explored through scanning electron microscopy (SEM). The results showed that high TEOS: MTES molar ratios improved the hydrophobicity of the superhydrophobic coatings, and the moisture resistance of the modified wood was improved. The coatings also exhibited good heat resistance and adhesion to wood [83]. Natural plant polyphenols also exhibit potential for the preparation of superhydrophobic coatings. Liu et al. prepared a three-dimensional-network-containing functional coating (namely, MPN) by chelating plant polyphenols with metal ions (Figure 4a). During synthesis, rutin, the main component of wood polyphenols, was used as the raw material for chelation with various metal ions (such as Al(III) and Fe(II)) to prepare the MPN. The MPN was then applied to the wood surface to generate a micro-/nanoscale rough layered coating. The coating exhibited excellent superhydrophobicity, self-cleaning ability, mechanical durability, acid and alkali resistances, and antifouling performance, and offered remarkable application prospects in building and decorative materials [84].

The sol–gel method has no special requirements pertaining to the material and area of substrates. The method is simple, its reaction conditions are mild, and the involved reactants can be evenly dispersed in a short period. The method is suitable for large-scale preparation, which can be expanded, and is commonly used for preparing superhydrophobic wood surfaces. However, the sol–gel method has the problems of a high raw-material cost, long processing time, and low preparation efficiency, as well as the generation of numerous micropores in the formed gel, which allows the escape of gases and organic substances during curing, leading to the formation of uneven superhydrophobic coatings [85].

### 3.2. Hydrothermal Method

The hydrophobicity of most wooden materials is observed only on their surfaces, and when the internal structure is exposed, the hydrophobic effect weakens or even disappears. Therefore, the development of construction schemes that consider superhydrophobic properties on the surface and in the internal structure of wood has gained importance [86]. The hydrothermal method creates a high-temperature and high-pressure environment using an aqueous solution as the reaction medium. Substances that are insoluble or difficult to dissolve under atmospheric conditions are dissolved under high-temperature and high-pressure conditions to achieve a certain degree of supersaturation. This condition causes crystallization and growth on the matrix surface that lead to the formation of micro-/nanoscale rough structures [87,88]. Among numerous methods for the construction of superhydrophobic structures in wood, many scholars have used hydrothermal methods to improve the surface roughness of wood and modify it with low-surface-energy substances. Liu et al. used a hydrothermal method to prepare superhydrophobic-wood-based composites. In particular, ammonium nitrate and tetrabutyl titanate were mixed in a certain proportion and shaken well. Then, eucalyptus samples were placed in the mixed solution and heated. Wood-based composites with superhydrophobic or superoleophilic properties were successfully synthesized under hydrothermal conditions. Experimental findings revealed that Ti and Si particles successfully entered the internal structure of wood and formed a superhydrophobic layer with a rough structure. The corresponding static contact angle reached 153°, and the rolling angle was very low. Thus, the eucalyptus sample surfaces showed excellent superhydrophobic properties [89]. In practical applications, block-shaped superhydrophobic wood exhibits high durability and wear resistance. However, the manufacturing process of block-shaped superhydrophobic wood still faces significant challenges, especially in achieving superhydrophobic effects in the internal structure of wood. Tan et al. synthesized block-shaped superhydrophobic wood through a combination of vacuum impregnation and hydrothermal methods (Figure 4b). First, zinc oxide nanorods were prepared in situ on the surface and interior of wood. Then, hydroxyl groups in zinc oxide nanorods and wood were replaced by long-chain alkyl groups via a treatment, which resulted in lowered surface energy of the wood and the formation of micro-/nanoscale rough structures. Analysis of elements and morphological structure of the wood revealed the presence of micro-/nanostructures and their hydrophobicity. After the treatment involving replacement by long-chain alkyl groups, the contact angle reached up to 155°. After 180 s of corrosion and sawing, the contact angle of the wood could still reach 150°, which indicated that the internal structure of the wood showed superhydrophobic properties. After treatment with various chemical reagents, the contact angle could be maintained at approximately 150°, which indicated that the modified wood possessed excellent superhydrophobicity and chemical durability [90].

The hydrothermal method is simple to operate and can be used to synthesize various superhydrophobic NPs through alterations of the reaction conditions. Moreover, the size and morphology of the NPs (such as nanoflowers and nanorods) can be controlled. The hydrothermal method can also overcome the incapability of modifiers to enter the wood interior to a certain extent. However, the randomness of the in situ generation of NPs in a wood interior leads to the uneven distribution of micro-/nanostructures [91]. In summary, various NPs synthesized by the hydrothermal method can endow superhydrophobic wood with further functional and intelligent characteristics and thus possess enormous development potential and value. However, the hydrothermal method requires specialized high-pressure vessel equipment. During the synthesis of NPs, fluid volume in the reaction vessel increases, which can generate extremely high pressures. Thus, the equipment has extremely high requirements for temperature, pressure, and corrosion resistances. High-temperature and high-pressure conditions limit the preparation of large-area superhydrophobic coatings.

### 3.3. Template Method

The template method offers stronger controllability and more convenient operation compared to other preparation methods. The method involves the preparation of thin films with micro-/nanostructures through the extrusion of or coating of hydrophobic materials on templates with rough structures, which elicit superhydrophobic effects [92]. The route for the synthesis of superhydrophobic nanomaterials via the template method generally consists of three steps: (1) preparation of templates with a low surface energy, (2) use of the etching method to replicate rough structures under the action of templates to synthesize target products, and (3) removal of the templates to obtain superhydrophobic surfaces [93]. The selection of appropriate templates for nanomaterial preparation is crucial in the synthesis of micro-/nanoscale materials through the template method. Template types can generally be divided into two categories: the first type includes synthetic materials (porous materials, surfactants, and NPs), wherein liquid polymers, such as polytetrafluoroethylene and polydimethylsiloxane (PDMS), are mainly used as molding materials. These materials are poured onto surfaces with micro-/nanostructures and separated from the surfaces after curing, ultimately generating rough morphologies with micro-/nanostructures [94,95]. The other template type includes biomimetic natural materials (such as lotus leaves, water strider feet, and cicada wings). The superhydrophobic texture is mainly obtained through etching. The substrate is derived from superhydrophobic surfaces found in nature, such as leaves, insect wings, and reptile skin. By controlling the shape of the mold, desired array structures are imprinted on the substrate surface, which can be used to achieve low-cost, high-resolution, and high-throughput structural replication [96,97]. Wang et al. used lotus leaves as superhydrophobic templates, sealed them with PDMS, and replicated micro-/nanostructures observed on the surface of lotus leaves via the template method. Based on this, superhydrophobic coatings with excellent wear resistance were prepared through the addition of nano-SiO_2_ microspheres. The synthesized rough surface resembling a lotus leaf achieved a contact angle of 152.5°, which implied good dimensional stability and hydrophobicity [98]. Due to its anisotropy and porous structure, wood with vertical channels has often been used as a biological template to prepare superhydrophobic structures. Ma et al. proposed a novel self-assembly technique for oil–water separation. In particular, wood was treated with polyethyleneimine, sodium alginate, ammonium polyphosphate, and chitosan compounds. Next, the self-assembled wood template was immersed in a mixture of silica NPs and 3-aminopropyltriethoxysilane (APTES) to render the wood template with hydrophobicity (Figure 4c). In this process, APTES was used as a crosslinking agent and silica was used to construct micro-/nanostructures, which improved the mechanical stability, hydrophobicity, and chemical stability (repulsive effect toward corrosive liquids) of the wood biological template. The synthesized template material exhibited excellent flame retardancy, mechanical durability, and self-cleaning capability, along with a contact angle of up to 168° [99].

The template method can achieve the controllable regulation of the morphology, particle size, and structure of nanomaterials by controlling the nucleation and growth of crystals during preparation. This method offers the advantages of high repeatability and low cost, as well as the stable synthesis of superhydrophobic structures [100,101]. The main component of the template method is the template material, and after template removal through certain methods (such as high temperatures), the template structure can be retained in the substrate. However, there remain challenges in the commercial application of the template method, such as those regarding template removal during the synthesis of nanomaterials. The separation of templates and products can easily cause damage to nanotubes, nanowires, and hollow nanospheres. The preparation process of templates is complex, requires high precision, and takes a long time. Therefore, the further development and application of the template method is slow.

### 3.4. Surface-Coating Method

**Figure 5:**
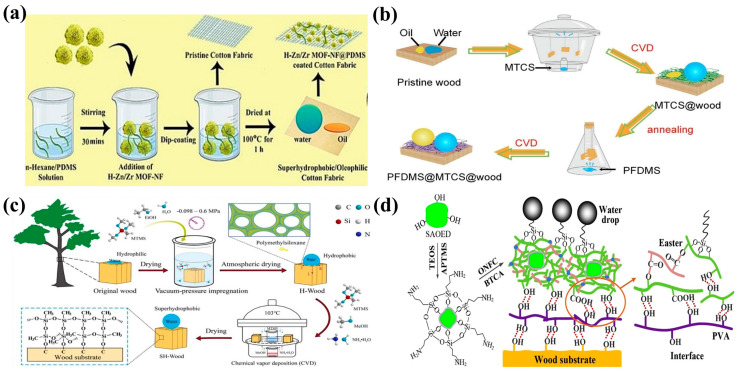
(**a**) Preparation of superhydrophobic cotton fabric and its application in oil−water separation [111]; (**b**) preparation of a superhydrophobic coating on a birch surface via the CVD [119]; (**c**) synthesis of superhydrophobic-wood-based composite materials via liquid-phase deposition [120]; (**d**) synthesis of superhydrophobic wood with a long afterglow [121].

The surface-coating method is used to prepare superhydrophobic coatings through spraying or deposition of low-surface-energy materials onto substrates (glass, wood, metal, textiles, etc.). This method can be applied to manufacture superhydrophobic coatings with various abilities, such as self-healing and corrosion resistance [102,103]. The surface-coating method mainly includes spray- and dip-coating methods. The spray-coating method disperses nanomaterials and low-surface-energy substances in a suitable solvent to generate uniform micro-/nanoparticles. The particles are then sprayed onto a substrate surface using specialized spraying equipment, and the surface particles are stacked to form a superhydrophobic surface with a micro-/nanoscale composite rough structure. After natural air drying or heating curing, superhydrophobic wood is obtained [104,105]. The basic principle of the dip-coating method is the immersion of a substrate in a prepared solution and the transfer of NPs to the substrate surface under the action of the surface tension of the liquid solution. After drying and curing, a superhydrophobic coating is formed on the substrate surface [106,107]. Factors such as solution type, soaking time, and modification additives can affect the hydrophobicity, adhesion, chemical stability, and thermal stability of the coating. In practical use, surface coating is generally used to apply paints and films on wood. Many paints and film-forming substances display hydrophobicity. Consequently, micro-/nanoscale rough structures can be constructed through the addition of other NPs to generate a superhydrophobic coating on the wood surface [108,109]. At present, the surface-coating method has been widely studied and applied in laboratory and industrial production. To solve the problems of the high preparation cost, complex manufacturing process, and poor durability of coatings prepared via the surface-coating method, Assem et al. employed the spraying technology to spray a nanocomposite material composed of silica and an epoxy resin onto a substrate surface to prepare a superhydrophobic coating with excellent hydrophobicity and durability. During the synthesis process, amino-functionalized PDMS was used for modification to overcome the high surface energy of the epoxy resin. The prepared nanocomposite coating exhibited excellent superhydrophobicity, with rolling and contact angles of 3° and 165°, respectively (Figure 4d). Further, the influence of the silica concentration on the coating effect was analyzed, and the results revealed that the composite coating prepared using 28 wt% silica achieved the best durability and superhydrophobicity. The influence of solvent volatility on the coating structure was also studied. The solvent with low volatility easily filled the microstructure, improving the coating hydrophobicity [110]. Oil spills threaten the aquatic environment and human health, and identifying effective oil–water separation methods is crucial to solving such environmental problems. Ahmad et al. developed a superhydrophobic cotton fabric for oil–water separation. They first synthesized a nanoflower-shaped bimetallic organic framework (H-Zn/Zr MOF-NF) and functionalized its surface using myristic acid (Figure 5a). Subsequently, PDMS and H-Zn/Zr MOF-NF were coated onto the surface of cotton fabric via dip coating to reduce surface energy and improve surface roughness, which rendered the fabric with superhydrophobicity. The rolling and contact angles of the functionalized cotton fabric were 4° and 161°, respectively, and the fabric showed high separation capability for oils, such as diesel and toluene, with a separation efficiency of up to 98%. After 12 cycles of adsorption–desorption, the contact angle could still reach 156°, indicating that the composite material possessed excellent superhydrophobicity and reusability. This study provides unlimited possibilities for the development of superhydrophobic materials in the field of oil–water separation based on the dip-coating method [111]. In daily life, spray coating is one of the commonly used methods for the preparation of superhydrophobic surfaces due to its easy operation, wide applicability, and easy large-scale use. However, this method requires strict control of the spray time, spray distance, particle concentration, and particle size to obtain ideal superhydrophobic coatings [112]. In addition, NPs sprayed onto the substrate surface display poor adhesion and are prone to detachment when subjected to physical scratching, which results in the loss of superhydrophobic properties. A certain proportion of adhesives (such as epoxy resins or acrylic polyurethane) is usually added to coating materials to increase the adhesion between NPs and the substrate surface, which improves coating durability and robustness [113]. The dip-coating method has the advantages of low cost and easy implementation. However, the coating thickness is affected by the ion concentration in the solution, which results in poor controllability of the thickness [114]. However, the coating thickness is affected by the ion concentration in the solution, which results in poor controllability of the thickness. Nevertheless, repeated coating at any time can quickly repair damaged superhydrophobic surfaces. Therefore, the preparation of superhydrophobic materials via the surface-coating method has broad application prospects.

### 3.5. Chemical Deposition Method

The preparation of superhydrophobic coatings via chemical deposition involves the deposition of low-surface-energy NPs onto a substrate surface through chemical reactions to generate a coating with a certain roughness to achieve superhydrophobicity. The method mainly includes chemical vapor deposition (CVD), liquid-phase deposition, and electrochemical deposition [115]. CVD involves the vaporization of reactants and their reaction with substrate materials to prepare precise rough structures through controlled reaction conditions. This method is commonly used to deposit superhydrophobic coatings or low-surface-energy substances onto substrates. If low-surface-energy substances are also used as reaction precursors and the reaction conditions are controlled to vaporize and deposit these substances onto the substrate surface together with the precursor for preparing rough structures, then the preparation steps can be simplified and superhydrophobic structures can be prepared in a single step [116]. The principle of preparing superhydrophobic coatings via liquid deposition involves the introduction of the precursor of a coating material into the solution and immersion of the substrate in the solution. Through a chemical reaction, the coating material is deposited onto the substrate [117]. Electrochemical deposition refers to coating formation on a substrate surface via electrochemical reactions of metal ions in a solution and subsequent modification using low-surface-energy substances to obtain a superhydrophobic surface with mechanical durability and corrosion resistance. Metal ions in the solution acquire electrons near the cathode, and the surface roughness and microstructure of the deposited layer can be controlled by controlling factors such as electrolyte composition, solution pH, and current density [118]. As shown in Figure 5b, superhydrophobic wood has great development potential in practical applications. Jian et al. coated 1H,1H,2H,2H-perfluorodecyltrimethoxysilane (PFDMS) and methyltrichlorosilane (MTCS) onto the surface of birch wood via the CVD method. The resultant PFDMS@MTCS coating exhibited excellent superhydrophobicity, chemical stability, and durability, along with a water contact angle of up to 157.7°. After soaking in strong alkaline, strong acidic, and organic solvents for 24 h, the contact angle remained unchanged. After prolonged mechanical friction, the coating retained its basic morphological structure, showing potential for long-term use in harsh environments [119]. Liquid-phase deposition also yields superhydrophobic-wood-based composite materials. Tang et al. prepared superhydrophobic wood with a low rolling angle (<10°) and a high contact angle (>150°) via the liquid-phase deposition method (Figure 5c). First, methyltrimethoxysilane (MTMS) was used to filled in wood cell walls through vacuum impregnation. Subsequently, MTMS was deposited onto the wood surface via the liquid-phase deposition method. The morphology and chemical composition of the resulting superhydrophobic wood were analyzed using SEM and energy dispersive X-ray spectroscopy, and it was found that MTMS was successfully grafted into the cell walls of the modified wood, which resulted in the formation of a dense PDMS protective layer on the wood surface. Modified materials have exhibited excellent superhydrophobicity, self-cleaning performance, durability, wear resistance, and decay resistance [120]. The synthesis process combining CVD and spray coating has also been used to prepare superhydrophobic coatings. Zhang et al. modified fluorescent powder using TEOS and 3-aminopropyltrimethoxysilane to synthesize a dual-function coating material with superhydrophobicity and luminescence by mixing the modified fluorescent powder with polyvinyl alcohol (Figure 5d). The coating material was applied on the wood surface, which showed a rolling angle of 8.5° and a water contact angle of 153°. The synthesized superhydrophobic wood with extended afterglow could be used in fields such as night indicators and interior decoration [121].

Compared with other methods, CVD shows a simpler preparation process, a lower cost, and more suitability for large-area thin-film preparation. This method can be used for substrates, such as wood, glass, metal, and textile substrates, and offers broad application prospects in metal rust prevention and corrosion resistance.

### 3.6. Vegetable Oil-Based Hydrophobic Protection Methods

Vegetable oil is widely distributed in nature and is extracted from the fruits, seeds, and germ of plants. The main component of vegetable oil is triglycerides, which are synthesized from glycerol and fatty acids and contain ester groups and unsaturated double bonds. Vegetable oil exhibits good hydrophobicity and thermal conductivity, making it a promising environmentally friendly wood modifier. At present, the main vegetable oils used for wood modification are linseed oil, soybean oil, canola oil, tall oil, palm oil, and coconut oil. A hydrophobic film formed by vegetable oil on the wood surface can cover hydroxyl groups on the wood surface and improve its surface wettability. For example, after being coated with tung oil, the spruce surface displayed a contact angle of >160° and superhydrophobic properties. This is because tung oil hindered water movement in the internal channels of wood, reducing the water absorption rate. Thus, the spruce surface exhibited a hydrophobic effect [122]. In addition, using linseed oil to modify wood is a natural method for wood protection. Linseed oil enters the internal conduits of wood and forms a stable flaxseed oil film on its pore surface, reducing its water absorption rate and increasing its biological tolerance. Compared with linseed oil, the hydrophobicity of linseed oil treated with epoxidation was considerably improved. Chen et al. used epoxy linseed oil and carnauba wax for the hydrophobic modification of natural wood. Carnauba wax is the hardest natural wax, with good hydrophobicity, which can effectively reduce the wood moisture content. A combination of epoxy linseed oil and carnauba wax has a synergistic effect, which can enhance the hydrophobic properties of natural wood and hinder its biodegradation by microorganisms. The composite lotion possessed excellent hydrophobicity [123].

In this section, we discussed in detail preparation methods for superhydrophobic-wood-based composite materials and analyzed comprehensively the applicability of different methods. In the synthesis of superhydrophobic coatings, the synergistic effect of micro-/nanoscale rough structures and low-surface-energy substances jointly determines the superhydrophobic properties of a material surface. When preparing superhydrophobic surfaces, reducing the surface free energy is easy at the technical level. The key is the construction of suitable micro-/nanoscale rough structures. Many techniques are used to construct rough surfaces, and Table 1 summarizes these common methods used for constructing superhydrophobic coatings on wooden materials.

## 4. Wettability Changes in Hydrophobic Wood During Prolonged and Continuous Contact with Aqueous Phases

When water droplets come in contact with superhydrophobic coatings on substrates such as metals or polymers, due to the special microstructure and low-surface-energy characteristics of superhydrophobic coatings, the contact angle between the water droplets and the coating surface is usually very large, exhibiting significant hydrophobic properties. However, the contact angle may undergo some changes through continuous contact with water. For metal substrates, their good thermal conductivity may affect the contact angle in environments with temperature changes. An increase in ambient temperature will lead to a gradual decrease in contact angle. In addition, metal surfaces may undergo oxidation or corrosion after long-term contact with liquids, altering the chemical properties of superhydrophobic surfaces and affecting the performance of superhydrophobic coatings, resulting in a decrease in contact angle, as demonstrated in Kuzina’s work [124]. For polymer substrates, Klimov’s research indicated that the weak mechanical strength and chemical stability of polymers were key factors affecting the contact angle. Long-term continuous contact may cause a certain degree of deformation or aging of the polymer substrate, affecting the microstructure of the coating, thereby reducing its hydrophobicity and decreasing the contact angle. Moreover, polymer substrates may swell or degrade under the action of certain solvents or chemicals, which can also have adverse effects on the contact angle of superhydrophobic coatings [125].

In addition, whether the substrate is metal or polymer, pollutants, dust, and other impurities in the environment may adhere to the coating surface during long-term contact, damaging its low-surface-energy characteristics and gradually reducing the contact angle. As a substrate, the superhydrophobic coating on the surface of wood exhibits significant differences in contact angle behavior compared to other substrates such as metal, plastic, glass, etc.

During prolonged and continuous contact with aqueous phases, the wettability of hydrophobic wood undergoes a series of changes, which are mainly divided into the following stages: (a) during the initial contact between wood and water, hydrophobic groups are introduced on the wood surface through hydrophobic treatment or hydrophobic components are present in the wood extract, resulting in a larger contact angle of water droplets on the wood surface. The wood surface exhibits excellent hydrophobic effects, while the water absorption rate of wood is relatively low. (b) With prolonged contact of wood with water, water molecules gradually interact with hydrophobic groups or micro–nano rough structures on the wood surface. During this process, water droplets can damage or interfere with the hydrophobic layer on the wood surface, causing the contact angle of the wood surface to gradually decrease, while the water affinity of the wood surface is gradually improved. Therefore, more water molecules can enter the interior of the wood, gradually increasing its water absorption rate. However, the increase in the water absorption rate gradually slows down because the pore structure and cell walls inside the wood hinder water absorption to some extent [126]. (c) When hydrophobic wood comes into continuous contact with water for a long time, its wettability gradually tends to reach a stable state. Then, its water absorption rate is close to saturation, and the pores and cell cavities inside the wood are almost filled with water molecules, with the wood reaching its maximum water absorption capacity [127]. However, prolonged and continuous contact with water may further damage the hydrophobic structure of the wood surface, such as its coating peeling off, and hydrophobic components in wood may be dissolved, reducing its hydrophobicity and even causing it to lose its hydrophobic properties and be completely wetted by water. Meanwhile, the mechanical properties of wood may also be affected, leading to problems such as decreased strength and deformation [128,129].

Wettability changes on the wood surface are influenced by many factors. Owing to differences in its chemical composition, microstructure, etc., different woods exhibit varying rates of hydrophobicity and wettability changes in water. For example, wood containing high amounts of resin, wax, and other extracts may exhibit good hydrophobicity and can maintain its hydrophobic effect in water for a long time. Meanwhile, changes in the wettability of wood treated with different hydrophobic modification methods will also vary. For example, wood modified with nanocoating technology may have stabler hydrophobic properties, with relatively few changes in wettability when in contact with water for a long time. However, wood coatings formed via simple surface-coating methods may be more prone to detachment under the action of water, resulting in significant changes in wettability. In addition, the temperature, acidity, salinity, and microorganisms contained in water can affect the wettability of hydrophobic wood. Generally, high temperatures, strong acid or alkali environments, high salinity, and abundant microorganisms will accelerate the deterioration of the hydrophobic structure on the wood surface, causing rapid changes in the wettability of the wood [130].

Overall, superhydrophobic coating on the surface of wood has excellent stability, and synthesized superhydrophobic wood has a wide range of application prospects. The next section summarizes the application progress and future development directions for superhydrophobic wood in oil–water separation, self-cleaning, flame retardancy, and self-healing applications, providing useful references for future research.

## 5. Application of Superhydrophobic Wood

As per previous discussions and reports, superhydrophobic wood has attracted widespread attention recently due to its enormous application potential. In addition to hydrophobic properties, other functions of superhydrophobic wood need consideration in practical applications. For example, superhydrophobic wood used in building structures requires properties such as decay resistance, antibacterial properties, aging resistance, and flame retardancy [131]. Superhydrophobic wood used in indoor decoration needs to show abilities such as dust-prevention, self-cleaning, and oil-repellency abilities [132]. Superhydrophobic wood used in the transportation field needs to have anti-icing and anti-freezing capabilities [133]. During preparation, low-surface-energy materials and micro-/nanostructures exhibit various properties, including magnetism, conductivity, self-cleaning ability, microwave absorption, self-healing ability, and superhydrophobicity–superhydrophilicity transformation. These properties greatly promote the development and application of superhydrophobic wood [134,135,136]. Therefore, it is crucial to explore the application potential of effective strategies for preparing multifunctional superhydrophobic wood showing anticorrosion ability, UV resistance, and other abilities during oil–water separation, self-cleaning, flame-retardancy, and self-healing applications, among others. The following content provides a summary of the latest research work on superhydrophobic wood used in practical applications.

### 5.1. Oil–Water Separation Field

With increasing global industrialization, industries such as crude oil production, petrochemical, textile, leather processing, and food processing generate a large amount of oily wastewater, which is the most common pollutant. In addition, frequent oil spills in the sea have caused serious pollution and energy waste. To treat oil–water mixtures and achieve oil–water separation, researchers have adopted various treatment methods, such as porous carbon material adsorption, metal mesh filtration, and chemical oxidation treatment [137,138]. However, despite progress in these areas, many problems remain, such as high costs, low efficiency, difficulty in biodegradation or recycling, limited ability to handle different pollutants, complicated processes, difficult-to-maintain systems, and potential secondary harm to the environment [139]. Therefore, more efforts are urgently needed to build low-cost and efficient materials to achieve sustainable separation of oil–water mixtures. Wood is a sustainable biomass material with advantages such as biodegradability, a distinct hierarchical structure, abundant hydroxyl groups on its surface, a rich pore structure, high mechanical strength, and low cost. This material also exhibits anisotropy, drying shrinkage, and wetting expansion. These characteristics provide natural conditions for efficient oil–water separation. Therefore, the development of efficient wood-based oil–water separation materials has practical significance.

**Figure 6:**
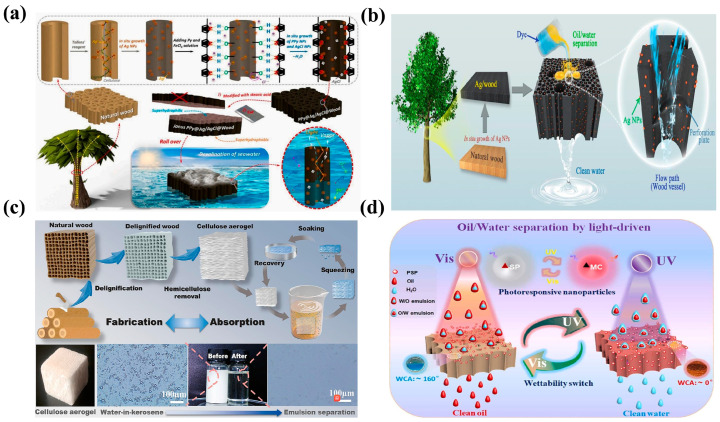
(**a**) Application of a Janus mesoporous wooden filtration membrane in oil–water separation [143]; (**b**) Ag/wood filter capable of simultaneously separating oil and organic pollutants [144]; (**c**) layered cellulose-based aerogels with an excellent oil–water separation performance [147]; (**d**) wooden sponge with a strong compression rebound effect that showed excellent durability, cyclic stability, and photoresponsive effects [149].

Based on various separation methods of oil from oil–water mixtures, hydrophobic and oleophilic oil–water separation materials can be divided into filter-type, adsorption-type, and intelligent-response-type oil–water separation materials. Filtration oil–water separation mainly involves selective oil–water separation when oil–water mixtures come into contact with the surface of filter-type oil–water separation materials due to the hydrophobic and oleophilic properties of the latter. Filtration oil–water separation usually employs sponge, metal meshes, synthetic membranes, and fabric as base materials. The surfaces of these base materials are modified via spraying, the sol–gel method, CVD, and other surface modification methods to generate micro-/nanoscale rough structures [140,141]. With wood serving as the template, a filter-type wood-based porous oil–water separation material is prepared by controlling the wettability of the wood surface and its internal hierarchical porous structure. This material selectively and efficiently filters out the water or oil phase from oil–water mixtures [142]. Numerous scholars have conducted research on wood-based oil–water separation materials, mainly involving the functional modification of wood, adjustment of its surface chemical properties, and improvement of its affinity for oils or water, to effectively promote oil–water separation. Directional filtration and transportation of oil–water mixtures are crucial for seawater desalination and oil–water separation (Figure 6a). Zhang et al. prepared a novel Janus mesoporous wooden filtration membrane and analyzed its application capabilities for seawater desalination, dye removal, and oil–water separation. Given its special asymmetric wettability, the synergistic effects of polypyrrole and Ag/AgCl NPs, and the transpiration mechanism of trees, the synthesized membrane exhibited excellent intrinsic driving force and filtration capacity (the oil-absorption capacity reached eight times the membrane weight). In addition, the degradation efficiency of MB and RhB reached 86.4% and 91.9%, respectively. Furthermore, the membrane displayed excellent cyclic stability, acid and alkali resistances, and self-desalting capability. This design scheme paved the way for the development of photothermal materials with a high solar energy conversion efficiency, which can aid in the alleviation of water scarcity [143]. In practical applications, the development of high-performance and multifunctional filtration materials is urgent in the field of oil–water separation. Cheng et al. used wood conduits as an ideal support material for filtration materials and deposited Ag NPs in situ inside the channels of the conduits to develop a wood filter that can simultaneously separate oil and organic pollutants (Figure 6b). In particular, wood chips were first vacuum-immersed in a silver ammonia solution to distribute silver ammonia ions on the surface of the pores of the chips. Then, in situ reduction was carried out by heating to obtain a bifunctional silver–wood filter with Ag NPs fixed on the surface. The prepared Ag/wood filter could effectively separate oil–water mixtures, with a separation efficiency of up to 99%. Given the catalytic activity of Ag NPs deposited inside the wood chips, MB could be efficiently removed during oil–water separation. The removal efficiency of MB was closely related to the thickness of the Ag/wood filter [144].

Most adsorption-type oil–water separation materials utilize the hierarchical porous structure of materials to achieve selective separation of oil and water. Given the hydrophobic and oleophilic properties of such materials, oil enters the materials through pores while water is blocked on the surface. During oil–water separation, the oil–water separation materials can usually recycle and process the absorbed oils. The methods commonly used to prepare adsorption-type oil–water separation materials include CVD, self-assembly, sol–gel, and template methods [145]. For wooden materials, the main approach involves the utilization of the rich pore structure as well as the chemical modification and structural control of wood to prepare adsorption-type porous oil–water separation materials. These materials normally exhibit environmental friendliness, have wide sources of raw materials, show excellent adsorption effects, and have broad application prospects. Cai et al. applied a simple two-step method to prepare wooden sponge oil–water separation materials with excellent oil-absorption capacity and sustainability [146]. First, natural wood was subjected to delignification and hemicellulose treatment, and then a wood sponge was prepared through a freeze–drying process. Subsequently, the wooden sponge was immersed in solutions containing polyvinyl alcohol and PDMS to obtain wooden sponge oil–water separation materials with excellent compression rebound performance and hydrophobicity. Results indicate that the sponge had a high elastic recovery rate (reaching approximately 100% after 200 cycles), excellent lipophilicity (oil-absorption capacity = 25 g·g^−1^), and hydrophobicity (contact angle = 138°). Lignocellulose-based aerogels have great application potential in the field of oil–water separation. Wu et al. designed an efficient and recyclable oil–water separation material that showed fast and continuous operation (Figure 6c). First, hydrophilic cellulose nanofibers were obtained by removing some amount of hemicellulose and lignin from natural wood. After freeze–drying, cellulose-based aerogels with a layered structure, which provided a large space for oil storage and transportation, were successfully synthesized. The aerogels showed excellent oil–water separation capability and a separation efficiency of 99.97%. The aerogels maintained a stable performance despite exposure to different external environments [147].

Intelligent responsive materials are often used in oil–water separation. The construction of intelligent responsive oil–water separation materials mainly takes advantage of the special interfacial wetting characteristics of materials to convert oil–water separation methods under various environmental conditions and achieve separation and filtration of water and oils. Du et al. selectively removed hemicellulose and lignin from balsa and prepared a wooden sponge with a strong compression rebound effect via freeze–drying. Further, a pH-responsive polymer was synthesized through free-radical polymerization using 2-(dimethylamino)ethyl methacrylate and lauryl methacrylate as raw materials. Subsequently, the pH-responsive polymer was coated onto the surface of the synthesized wooden sponge via spray coating to prepare a pH-responsive superhydrophobic wooden sponge with an excellent oil–water separation effect. Results showed that the wettability (hydrophobicity and hydrophilicity) of the surface of the wooden sponge varied with changes in the pH level. The oil–water separation efficiency of the sponge reached 92%, and the sponge could be continuously reused (with excellent oil-absorption performance even after multiple mechanical squeezes) [148]. The optimization and utilization of wooden structures and components can be enhanced to achieve the efficient and sustainable treatment of oily wastewater using wood-based porous materials. Wu et al. performed free-radical polymerization to prepare a poly-spiropyran–basswood (PSP/BW) filtration membrane with surface wettability conversion capability. The membrane could realize the conversion of its hydrophilic and hydrophobic properties under varying light conditions (Figure 6d). It achieved an excellent oil–water separation effect, with a separation efficiency of up to 99%. It exhibited excellent durability, cycling stability, and photoresponsive effects [149].

In summary, the hierarchical structure, abundant pores, and easy chemical modification of wood make it ideal for oil and water separation, whether as a filter, an adsorbent, or a smart response material. Therefore, in future research, the diversified utilization of wood structures must be enhanced to achieve the efficient treatment of oily wastewater.

### 5.2. Self-Cleaning Ability

The use of superhydrophobic coatings in self-cleaning applications has always been a research hotspot, and the unique micro-/nanostructures of the lotus leaf surface provide it with an excellent self-cleaning ability. When a liquid contacts the micro-/nanostructures of this superhydrophobic surface, the liquid droplets easily roll off from the superhydrophobic surface and remove dust, dirt, and bacteria present on the surface due to the formation of a solid–liquid–gas composite interface, indicating self-cleaning characteristics. When wood is used in applications, a large amount of dust adheres to the surface due to static electricity. After eliciting superhydrophobicity in wood, spherically shaped water droplets form on the superhydrophobic wood surface and easily roll off, which results in the removal of dust from the wood surface and maintenance of its cleanliness. In addition, the self-cleaning ability of superhydrophobic wood is observed for other common liquids, such as cola, tea, milk, and coffee [150]. Researchers have prepared superhydrophobic surfaces for self-cleaning applications through various methods. Xia et al. synthesized a superhydrophobic coating with an excellent self-cleaning ability on a wood surface via a two-step method, achieving rolling and contact angles of 6° and 159°, respectively. In particular, SiO_2_ micro-/nanostructures were first constructed on a wood surface. Subsequently, a superhydrophobic coating was prepared through the modification of the structure using low-surface-energy substances (1H,1H,2H,2H-perfluoroalkyltrichlorosilane). The synthesized superhydrophobic coating was soaked in ink, and after a certain period, no black ink was observed on the wood surface, which indicates its self-cleaning ability. In addition, the coating exhibited good wear resistance, thermal stability, and acid and alkali resistances, which considerably improved the physical properties of the wood [151]. Wang et al. constructed Ti–Si nanoscale rough structures on the wood surface and modified them with PDMS to elicit a synergistic superhydrophobic effect for the synthesis of superhydrophobic wood with self-cleaning ability. Notably, the modifier in the coating could achieve superhydrophobicity and self-healing under thermal responses if the superhydrophobic surface was damaged. The synthesized superhydrophobic wood exhibited self-cleaning ability, could easily remove chalk dust and oleic acid from the wood surface, and showed good wettability [152].

**Figure 7:**
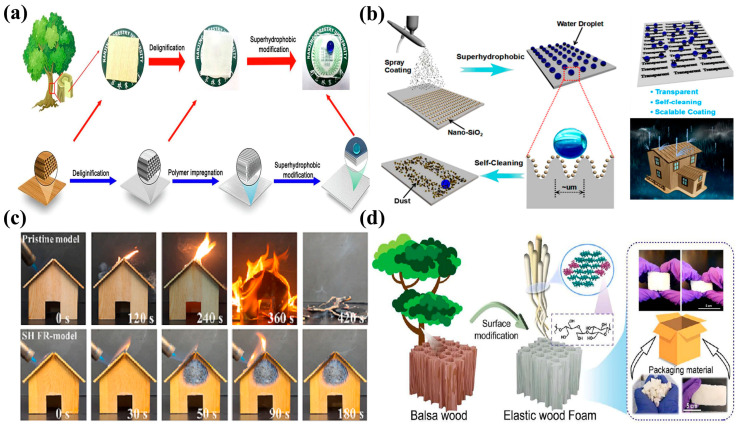
(**a**) Application of superhydrophobic transparent wood in antifouling and self-cleaning [153]; (**b**) preparation of cellulose-based transparent superhydrophobic coating [154]; (**c**) application of superhydrophobic flame-retardant coating on a wood surface [156]; (**d**) highly elastic wood sponge with excellent flame-retardancy properties [157].

Transparent self-cleaning wood can be used in the window surfaces of buildings. Manual cleaning of high-rise buildings is difficult and costly when dust accumulates, leading to decreased light transmittance. The use of superhydrophobic wood with self-cleaning ability and excellent light transmittance can reduce maintenance costs and the absorption of solar energy, achieving radiative cooling. Wu et al. analyzed the possible application of superhydrophobic transparent wood in antifouling applications (Figure 7a). First, wood was treated with hypochlorite to obtain delignified wood (TW). Then, the TW surface was modified using hydrophobic silica and a silane coupling agent to prepare a superhydrophobic transparent wood (STW), which had a thickness of 2 mm and excellent transmittance under visible light (93.7%), with self-cleaning ability. The STW was soaked in juice, mud, methyl orange, and methylene blue dyes, and the pollutants did not adhere considerably to the STW surface, which indicated the remarkable resistance of the wood toward pollutants [153]. Wei et al. used a top-down strategy to synthesize a cellulose-based transparent superhydrophobic coating (Figure 7b). The synthesized transparent coating had a rolling angle of less than 10° and a contact angle of greater than 150°. The superhydrophobic coating exhibited excellent optical reflection properties and good light transmittance as well as self-cleaning ability, which reduced the effect of dust accumulation on reflectivity [154].

### 5.3. Flame-Retardant Effect

Wood is a combustible biomass material with a low oxygen index value. When wood burns in the atmosphere, its flames quickly spread, which causes safety hazards to life and property. Thus far, fire accidents caused by wood storage and stacking and wood structure construction occur frequently. Many researchers have conducted exploratory work in this area, and they have been constantly seeking new nontoxic green flame retardants [155]. With the continuous development of nanoscience and technology, introducing nanofillers such as ZnO, SiO_2_, TiO_2_, and Mg(OH)_2_, which exhibit hydrophobic effects, and Al(OH)_3_, which has hydrophilic characteristics, into superhydrophobic structures can yield superhydrophobic wood with flame-retardant properties. Sun et al. used PDMS, flame retardants, and modified SiO_2_ NPs to construct a superhydrophobic flame-retardant coating, and it was applied to a wood surface. The ultimate oxygen index of the superhydrophobic flame-retardant wood increased by 31%, and the heat release rate decreased by 24.4%. The contact angle was greater than 154°, and the rolling angle was less than 5°, which implied excellent superhydrophobic effects and flame-retardancy performance (Figure 7c). In complex external environments (involving UV radiations, chemical corrosion, high temperatures, high humidity levels, mechanical impacts, etc.), the superhydrophobic flame-retardant wood also showed high weather resistance, good antibacterial effects, and chemical corrosion resistance [156]. Shi et al. applied a top-down strategy for the selective transformation of wood into highly elastic wood sponges (Figure 7d). On this basis, a continuous oxidation and reduction treatment process was adopted to synthesize lightweight and high-elasticity multifunctional materials (thermal insulation, flame retardancy, anisotropy, hydrophobicity, and high resilience). The material satisfactorily displayed an excellent flame resistance after the modification with organosilane [157]. In addition, transparent wood with flame-retardancy and superhydrophobic functions has been widely addressed in the field of construction. Aldalbahi et al. used ammonium polyphosphate, methyl methacrylate, and lanthanide-doped NPs to synthesize materials with flame retardancy, superhydrophobicity, UV shielding, and durable phosphorescence. After compounding the materials with transparent wood, the wood-based composite intelligent responsive material with a fluorescent effect was successfully synthesized. Under various environmental conditions, this responsive material exhibited different colors and thus has potential application prospects in fields such as outdoor lighting and smart windows [158].

In summary, through the comprehensive consideration of the current status and development trends of wood functional improvement, future research should involve the combination of flame retardancy and superhydrophobicity to attain progress toward multi-efficiency and environmental protection. The research on inorganic nanoprotection and the functional improvement of wood surfaces will be one of the important development directions of wood science in the future.

### 5.4. Self-Healing Properties

**Figure 8:**
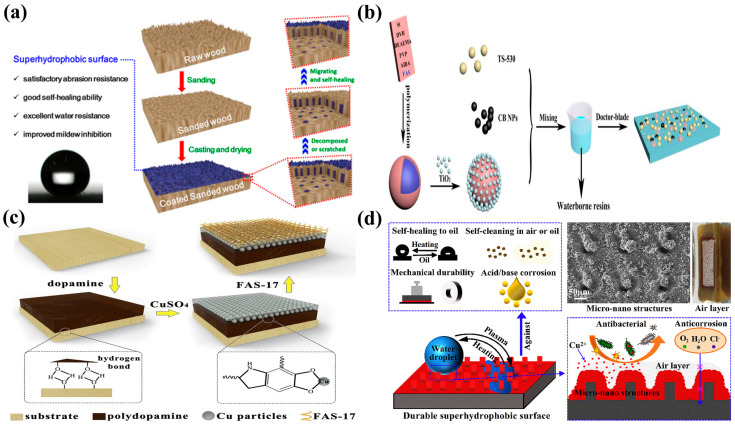
(**a**) Superhydrophobic coating modified with silica and fluoroalkyl silane exhibited a self-healing effect [47]; (**b**) synthesis of self-healing superhydrophobic coatings with NIR and UV light response [160]; (**c**) preparation and application of superhydrophobic antibacterial wood veneer [168]; (**d**) preparation of superhydrophobic antibacterial coatings via laser etching and spraying methods [169].

Superhydrophobic coatings have broad application prospects in oil–water separation, self-cleaning, anticorrosion, and flame-retardancy fields. However, in actual use, the coating surface is prone to varying degrees of physical or chemical damage, which can harm the constructed micro-/nanostructures or reduce the levels of low-surface-energy substances in the coating and subsequently result in a decreased hydrophobic effect of the coating. This condition greatly limits the practical applications of superhydrophobic materials [159]. To improve the durability and stability of superhydrophobic materials, some researchers took inspiration from self-healing materials and endowed superhydrophobic surfaces with self-healing properties by mimicking natural life systems. In this process, self-healing properties are introduced into the structure of superhydrophobic materials to produce reusable self-healing superhydrophobic components. This construction strategy, which is an effective means to solving the problem of nondurable superhydrophobic surfaces, can extend the service life of superhydrophobic materials and reduce maintenance costs at the later stage. Wang et al. used sandpaper to polish a wood surface to produce satisfactory micro-/nanoscale rough structures. On this basis, silica and fluoroalkyl silane were mixed and sprayed onto the wood to prepare a superhydrophobic coating (Figure 8a). The superhydrophobic coating exhibited excellent wear resistance. After 45 cycles of wear, the coating still exhibited good superhydrophobic properties. When the coating surface was damaged, given its self-healing properties, the superhydrophobic performance of the coating gradually recovered at room temperature. This superhydrophobic construction strategy can extend the service life of wood [44]. Microcapsule technology is also an effective mode of achieving self-healing performance. This technology uses nano- or microscale carrier containers to encapsulate various functional materials and controls the release of compounds to prevent functional materials from being affected by environmental factors such as temperature, light, oxygen, and water. When the material surface is damaged, the repair agent inside the material can be released by changing conditions, such as light, temperature, and pH. Subsequently, the repair agent migrates to the damaged area of the material, repairs its damaged surface, and restores the superhydrophobic properties of the material. Given the increase in the maturity of microcapsule technology in self-healing applications, such as wood and metal anticorrosion and concrete cracking repair, researchers have proposed the idea of using microcapsules to encapsulate repair agents and embedding them in superhydrophobic materials to prepare superhydrophobic self-healing coatings. Li et al. successfully synthesized a self-healing superhydrophobic coating with near-infrared (NIR) and (UV) response by mixing carbon black NPs, microcapsules, and silicone rubber (Figure 8b). When the coating was damaged by the external environment, the dual response of UV-NIR could quickly repair the damaged area. This self-healing superhydrophobic coating also displayed corrosion and wear resistance [160]. In addition, epoxy resin coatings have excellent adhesion and chemical stability and a wide range of application advantages in daily life. However, such coatings are prone to cracking and other phenomena under the action of a mechanical force. In addition, they have poor weather resistance and are easily affected by external factors such as ultraviolet light. Therefore, functionalizing epoxy resin coatings is beneficial for expanding their application scope. Hasan et al. prepared composite hydrophobic coatings by mixing epoxy resin with hydrophobic silica and compared the durability, hydrophobicity, and self-cleaning performance of these coatings with different modifiers (ethyl acetate, dichloromethane, and *n*-butyl acetate). The results showed that the composite coatings modified with *n*-butyl acetate exhibited many advantages [161]. Accordingly, Pulikkalpamibil et al. synthesized a UV-triggered self-healing coating using silica nanoparticles, halloysite nanotubes, and epoxy resin and studied its self-healing effect. The results suggested that the self-healing coating had excellent self-healing effects under sunlight and possessed UV-shielding properties and thermal stability, indicating potential for a wide range of applications [162].

Overall, the current research on self-healing superhydrophobic coating materials is in the early stages. Future research should focus on in-depth analysis of the self-healing process and superhydrophobic mechanism of self-healing superhydrophobic coating materials to provide a theoretical basis for the functionalization and performance improvement of materials. In addition, further exploration of the self-healing effect of microcapsule superhydrophobic self-healing coatings in different practical engineering environments will expand the application fields of self-healing superhydrophobic coating materials; enable the development of multifunctional composite self-healing superhydrophobic materials; endow materials with more functional characteristics, such as magnetic, electrical, and temperature responsiveness; and further improve the practicality of materials.

### 5.5. Decay Resistance and Antibacterial Ability

Numerous bacteria and fungi exist in daily living environments, and they have a strong survival ability, fast reproduction, and transmission ability. Research has shown that preventing bacteria and fungi from adhering to the surface of materials can prevent their spread. However, if pollution sources on the material surface can be removed or eliminated, anticorrosion and antibacterial properties can be achieved. Superhydrophobic materials can resist adhesion and self-cleaning. Therefore, they have broad application prospects in the fields of antibacterial and anticorrosion [163].

Nanomodifiers used to synthesize superhydrophobic coatings have large volumes and surface areas; hence, they can provide active sites for antibacterial reactions. The synthesized superhydrophobic coatings exhibited antibacterial and fungicidal effects by releasing reactive oxygen species or metal ions at active sites [164]. Meanwhile, the surface roughness of superhydrophobic coatings is closely related to their antimicrobial activity. Rough areas at the superhydrophobic interface can generate a large number of air pockets, decreasing the anchoring points of microorganisms and further reducing their attachment [165]. Some superhydrophobic coatings with vertical structures had spikes on their surfaces. These spikes can penetrate the cell walls of microorganisms, causing the leakage of cell contents and resulting in microbial death [166]. Wood is easily degraded by decaying fungi, and anti-decay treatment is an effective means of avoiding or reducing the microbial decay and degradation of wood. In addition to the above factors, the main principles of superhydrophobic wood’s anticorrosion and antibacterial effects include the following: (a) the growth of decaying fungi requires the presence of water. Given its excellent hydrophobic effect, superhydrophobic wood can eliminate the water source needed by decaying fungi to survive. (b) Lignin, cellulose, and hemicellulose in wood can supply the nutrients needed for the growth of decaying fungi. The hydrophobic coating of superhydrophobic wood can prevent microorganisms from decomposing nutrients in wood and thus exert anti-decay and antibacterial effects. Superhydrophobic anti-decay and antibacterial wood has become a current research hotspot. Yao et al. used surface modification technology to treat wood, making it suitable for applications in the superhydrophobic antibacterial field. In particular, the wood was soaked in a stearic acid ester solution for superhydrophobic modification, followed by further coating with glycerol stearoyl ester to form a superhydrophobic surface with a micro-/nanolayered structure and synthesize superhydrophobic anti-decay and antibacterial wood with a contact angle of 159.2°. During the synthesis process, comparison was conducted on the antibacterial capability of hydrophobic and superhydrophobic wood surfaces, and infrared characterization and antibacterial experiments were conducted to confirm the antibacterial effect of superhydrophobic wood. The findings indicate that superhydrophobic wood can completely prevent fungal degradation of wood, and natural wood and hydrophobic wood are subject to fungal degradation to varying degrees, which implies the excellent antifungal effects of superhydrophobic wood [167]. In daily life, wood veneer exhibits easy water absorption, deformation, and fungal decay, which limit its further application. The development of superhydrophobic antibacterial veneer can expand its use in decorative building materials. Duan et al. deposited Cu NPs in coatings through dopamine polymerization reaction and metal chelation and used fluorosilane for hydrophobic modification; then, they ultimately successfully prepared a superhydrophobic antibacterial wood veneer (Figure 8c). The modified veneer displayed a contact angle of 155.7°, a rolling angle of up to 4°, and excellent antifungal activity, a self-cleaning effect, and acid and alkali resistance [168]. Similarly, superhydrophobic materials show good antibacterial properties against bacteria. Zhang et al. initially constructed a rough structure on the substrate surface through laser etching and then sprayed a mixed coating composed of hexadecyltrimethoxysilane, KH550-CuO, and epoxy resin onto the substrate surface (Figure 8d). During this process, the mechanical interlocking effect that formed between the coating and the rough surface could further prevent the coating from peeling off. The synthesized superhydrophobic coating exhibited a good antibacterial effect, and antibacterial experiments showed its effective inhibition of the activity of bacteria in contact with the coating surface. Moreover, the coating presented excellent mechanical durability (after 20 wear cycles, the coating retained superhydrophobic and corrosion-resistant properties), self-cleaning, self-healing, and chemical stability. The designed synthesis strategy offers the characteristics of environmental protection, high efficiency, and low cost, and has a broad application potential in outdoor applications [169]. Gao et al. applied a two-step synthesis strategy combining the hydrothermal method and a silver mirror reaction to construct Ag-TiO_2_ micro-/nanoscale rough structures composed of TiO_2_ NPs and silver nanocrystals on a wood surface. Subsequently, the rough surface was nano-modified with trimethoxysilane, and a superhydrophobic coating with a contact angle of 153.2° was successfully synthesized. The surface exhibited excellent photocatalytic activity under visible-light irradiation. The antibacterial experiment results revealed the excellent antibacterial effects of the modified superhydrophobic wood on Gram-negative and Gram-positive bacteria [170].

Therefore, the above findings indicate the wide use of superhydrophobic coatings in applications such as oil–water separation, self-cleaning, flame retardancy, self-healing, and decay resistance. The construction of superhydrophobic coatings on the surface of wooden materials is a promising strategy to promote the efficient utilization of wooden resources and extend their service life.

## 6. Summary and Future Prospects

Wood is a sustainable biomass material with a hierarchical porous structure and anisotropy, and it is widely used in daily life. The abundant hydroxyl groups on its surface result in its easy absorption of water. Moisture can cause wood to deform, rot, and show decreased strength. Research reports have shown that the construction of superhydrophobic surfaces on wood can effectively inhibit water infiltration, which improves wood service life. In addition, the superhydrophobic modification of the surface of wood endows it with good properties, such as waterproofing, stain resistance, anticorrosion, high-temperature resistance, and self-cleaning, which make it more valuable in fields such as antifouling, anti-decay, and oil–water separation. Based on this, we summarized research progress in superhydrophobic-wood-based materials in the past three years. We hope to combine traditional coating technology with high-tech nanotechnology by constructing micro-/nanoscale rough structures with superhydrophobic properties on a wood surface to obtain wood products with waterproof, stain-resistant, self-cleaning, and self-healing surfaces, which will greatly improve the application range of wood products.

In this article, we first introduced the macroscopic and microscopic structural characteristics of wood and observed that its hierarchical porous structure can lay the foundation for the preparation of superhydrophobic coatings. Next, three theories of superhydrophobic surface wetting were elaborated, and the micro-/nanostructure characteristics of superhydrophobic wood surfaces were analyzed. The principles and methods of preparing superhydrophobic surfaces were then summarized. Finally, a summary of the progress of superhydrophobic-wood-based materials in oil–water separation, self-cleaning, flame-retardancy, self-healing, and anti-decay applications was provided. Scholars have recently made great achievements in the basic wetting theory as well as in superhydrophobic-surface design principles, preparation methods, and application research. However, the functional modification technology lacks maturity and cannot be widely applied. Many challenges still need to be addressed to make superhydrophobic wood more in line with future development trends.

(1) Mechanical stability of superhydrophobic wood: the mechanical stability of superhydrophobic wood is mainly reflected by the interfacial bonding force between superhydrophobic coatings and wood, as well as by the mechanical strength of micro-/nanostructures on the surface of superhydrophobic wood. In the actual processing and use, the poor adhesion between superhydrophobic coatings and substrates can lead to coating detachment, and the micro-/nanostructures on the surface of superhydrophobic wood can be damaged by impact, friction, and other forces, potentially affecting the hydrophobic properties of superhydrophobic wood.

(2) Exploring the universal laws and related theories of the influence of substrate micro-/nanostructure, roughness, and chemical stability on hydrophilicity and hydrophobicity: self-healing technology must be further integrated with superhydrophobic properties to achieve synergistic effects and enable the construction of self-healing superhydrophobic coating materials with excellent functionality and economy.

(3) Functional modification of superhydrophobic wood: if superhydrophobic wood is expected to meet future development strategies and become a research hotspot, a better combination of superhydrophobic technology and functional modification must be developed. Through the combination of various preparation techniques—such as physical, chemical, biological, and mechanical techniques—with biomimetic processes, the continuous improvement in the bonding strength between hydrophobic layers and substrates, the simplification of wood production processes, a reduction in wood preparation costs, and an extension of wood product lifespan can be truly realized, increasing the value of superhydrophobic wood.

## Figures and Tables

**Table 1:** The characteristics of typical synthesis methods for superhydrophobic coatings on the surface of wood-based materials.

Synthetic Method	Comparison in Conditions and Characteristics		
	Process	Pros	Cons
Sol–gel method	The sol was converted into gel, and superhydrophobic material was formed after curing.	Easy to control; simple process.	Long reaction time; uneven coating.
Hydrothermal method	High-temperature and high-pressure environment; recrystallization process.	Adjustable size and morphology; easy doping.	Slow process; complex reaction conditions.
Template method	Synthesize templates with low surface energy; preparation of rough structure; removal of template.	High repeatability; low cost; structural stability.	Difficulty in removing templates; long processing time.
Surface coating method	Uniformly dispersed particles are sprayed or deposited on the surface of the substrate.	Low costing; simple process; wide application range.	Poor adhesion; poor control.
Chemical deposition	Through chemical reactions, low-surface-energy particles are deposited on the surface substrate to form high-performance coatings.	Easy to control; high efficiency.	Slow deposition rate; long processing time.
